# Supplementary material for: Receptor for Advanced Glycation End-Products Promotes Activation of Alveolar Macrophages through the NLRP3 Inflammasome/TXNIP Axis in Acute Lung Injury
Source: Int J Mol Sci. 2022 Oct 1;23(19):11659. doi: 10.3390/ijms231911659 (PMC9569658; doi:10.3390/ijms231911659)
Supplement: Supplementary file 1 [file ijms-23-11659-s001.zip › ijms-1870995-supplementary.pdf]

ONLINE DATA SUPPLEMENT

Receptor for Advanced Glycation End-Products and NLRP3 Inflammasome-driven Macrophage Activation in Acute Lung Injury

Woodys Lenga Ma Bonda, Marianne Fournet, Ruoyang Zhai, Jean Lutz, Raiko Blondonnet, Céline Bourgne, Charlotte Leclaire, Cécile Saint-Béat, Camille Theilliere, Corinne Belville, Damien Bouvier, Loic Blanchon, Marc Berger, Vincent Sapin, Matthieu Jabaudon

Supplementary figure legends

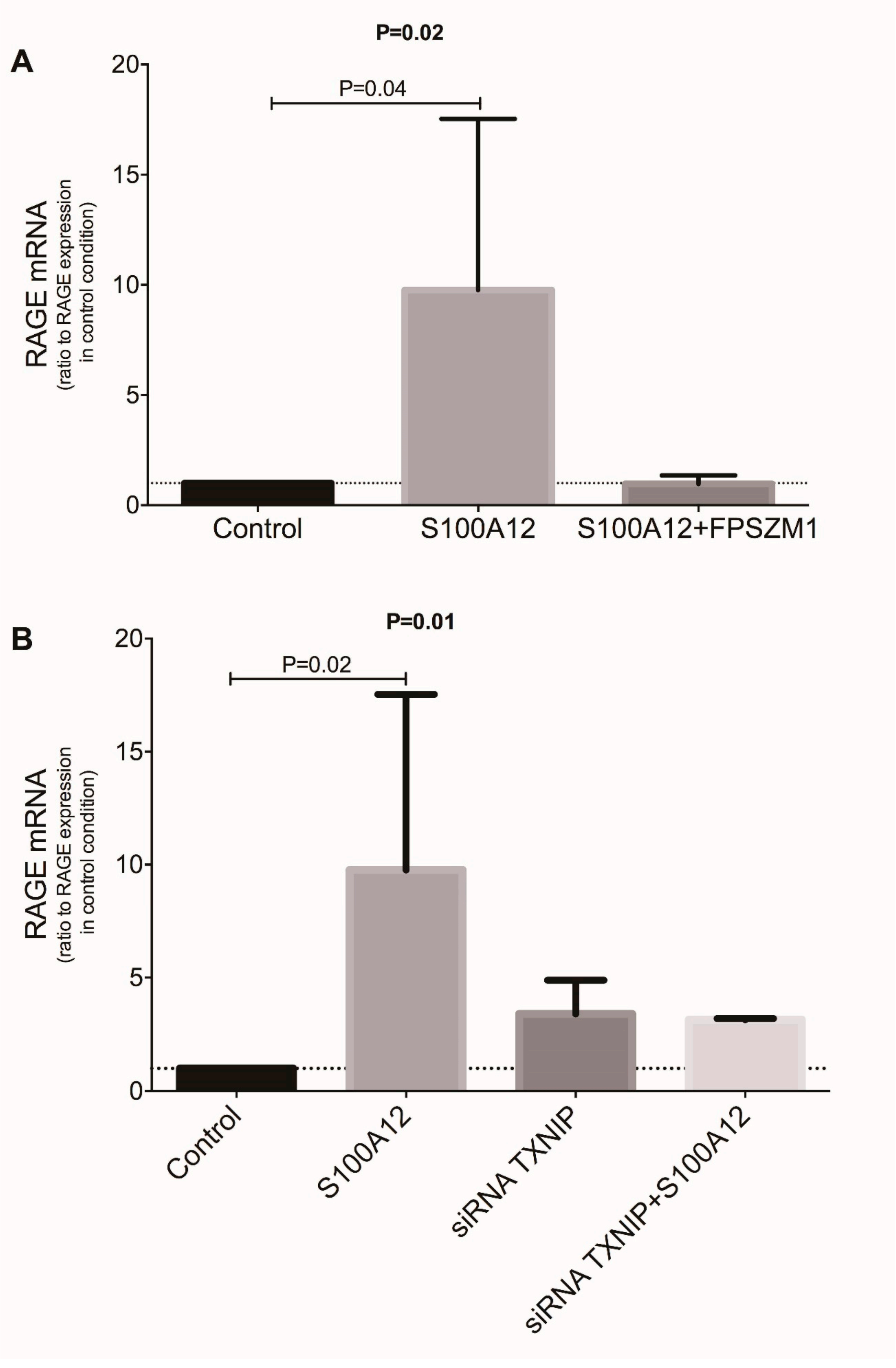

Figure S1. A) Levels of RAGE mRNA in THP-1 cells treated for 24 hours in the presence or absence of the RAGE agonist S100A12 (40 nM) (MyBiosource), alone or in combination with the RAGE antagonist FPS-ZM1 (1μM) (EMD Millipore), and **B)** in THP-1 cells transfected with specific small interfering (siRNA) oligonucleotides for mouse thioredoxin-interacting protein (TXNIP) (Dharmacon) and treated with S100A12 or phosphate-buffered saline (control). The efficiency of siRNA knockdown was verified using a duplicate enzyme-linked immunosorbent assay of TXNIP (CycLex Co.). Threshold levels of mRNA expression (ΔΔCt) were normalized to housekeeping genes. mRNA expression levels are expressed as ratios to those in the control condition (n = 6 in duplicate for each condition). Data are representative of three independent observations. Values are reported as means ± SD. The data were analyzed with the Kruskal–Wallis test (nonparametric data).

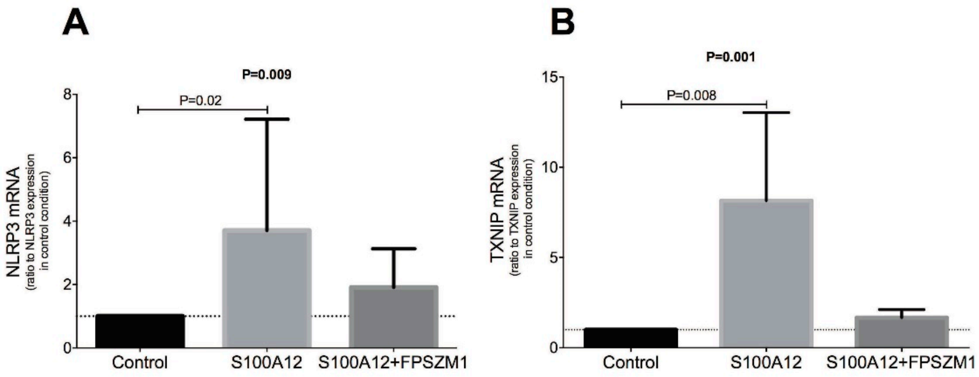

Figure S2. mRNA expression levels of **A)** NLRP3 and **B)** TXNIP in THP-1 cells treated for 24 hours in the presence or absence of the RAGE agonist S100A12 (40 nM) (MyBiosource), alone or in combination with the RAGE antagonist FPS-ZM1 (1μM) (EMD Millipore). Threshold levels of mRNA expression (ΔΔCt) were normalized to housekeeping genes. mRNA expression levels are expressed as ratios to those in the control condition (n = 6 in duplicate for each condition). Values are reported as means ± SD. The data were analyzed with the Kruskal–Wallis test (nonparametric data).

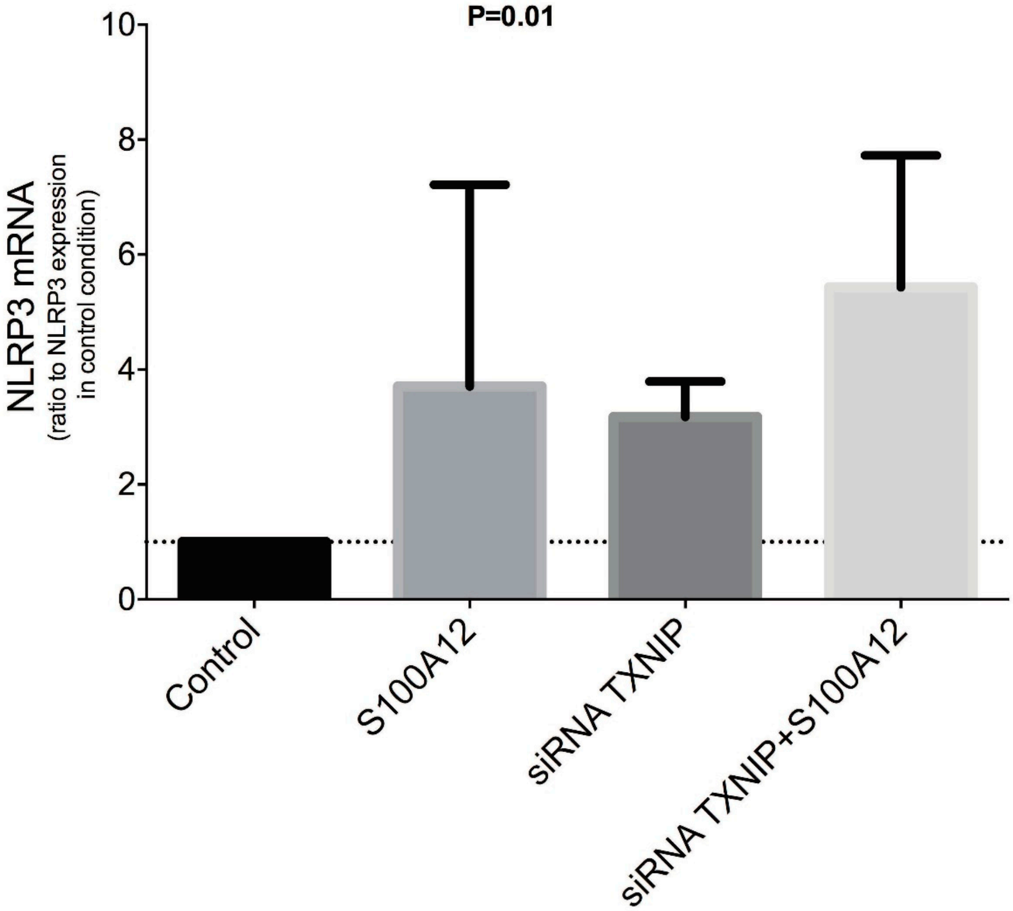

Figure S3. mRNA expression levels of NLRP3 in THP-1 cells transfected with specific small interfering (siRNA) oligonucleotides for mouse thioredoxin-interacting protein (TXNIP) (Dharmacon) and treated with S100A12 or phosphate-buffered saline (control). The efficiency of siRNA knockdown was verified using a duplicate enzyme-linked immunosorbent assay of TXNIP (CycLex Co.). Threshold levels of mRNA expression (ΔΔCt) were normalized to housekeeping genes. mRNA expression levels are expressed as ratios to those in the control condition (n = 6 in duplicate for each condition). Data are representative of three independent observations. Values are reported as means ± SD. The data were analyzed with the Kruskal–Wallis test (nonparametric data).

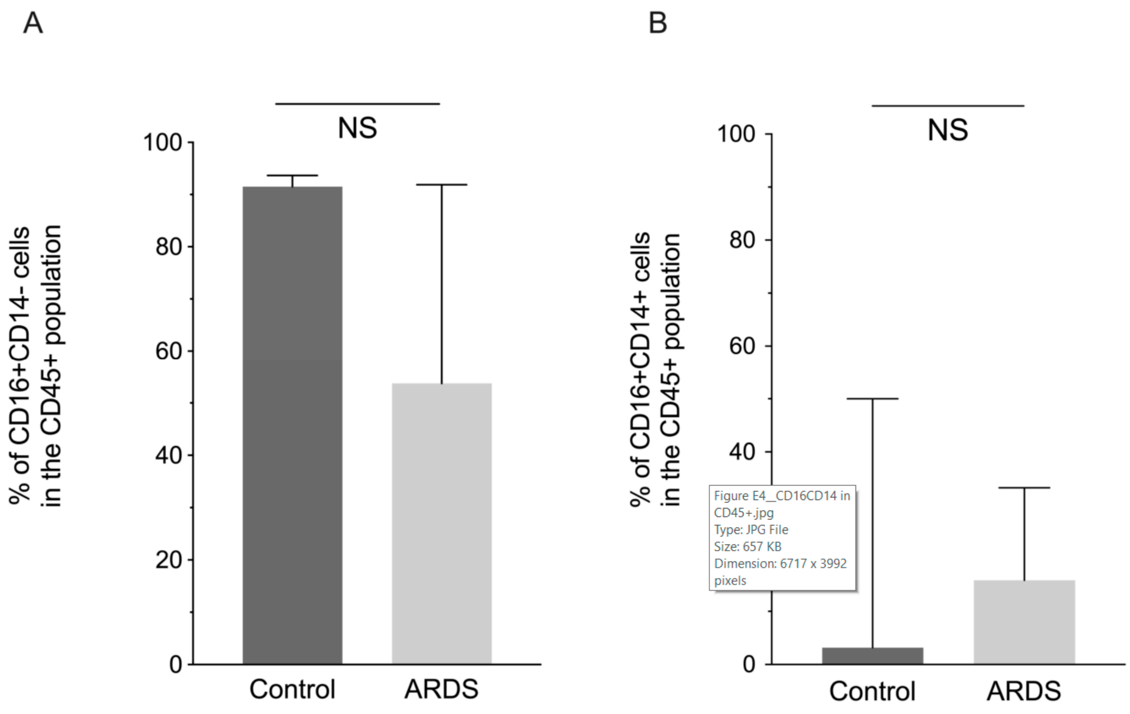

Figure S4. Percentages of **A)** CD16+CD14- cells in the CD45+ subpopulation and **B)** CD16+CD14- cells in the CD45+ subpopulation of alveolar macrophages from patients with acute respiratory distress syndrome (ARDS) and in mechanically ventilated patients without ARDS (control). Values are reported as medians ± interquartile ranges. The data were analyzed using the Mann–Whitney test (nonparametric data).

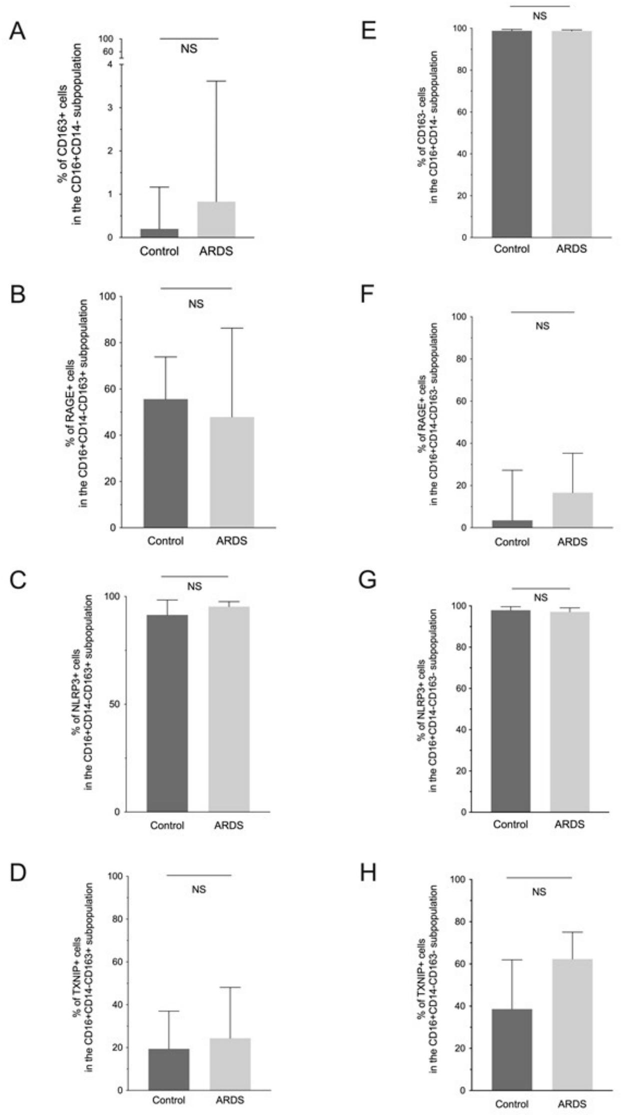

Figure S5. Percentages of **A)** CD163+ cells in the CD16+CD14- subpopulation, **B)** RAGE+ cells in the CD16+CD14-CD163+ subpopulation, **C)** NLRP3+ cells in the CD16+CD14-CD163+ subpopulation, **D)** TXNIP+ cells in the CD16+CD14-CD163+ subpopulation, **E)** CD163- cells in the CD16+CD14- subpopulation, **F)** RAGE+ cells in the CD16+CD14-CD163- subpopulation, **G)** NLRP3+ cells in the CD16+CD14-CD163- subpopulation, and **H)** TXNIP+ cells in the CD16+CD14-CD163- subpopulation of alveolar macrophages from patients with ARDS and in mechanically ventilated patients without ARDS (control). Values are reported as medians ± interquartile ranges. The data were analyzed using the Mann–Whitney test (nonparametric data).

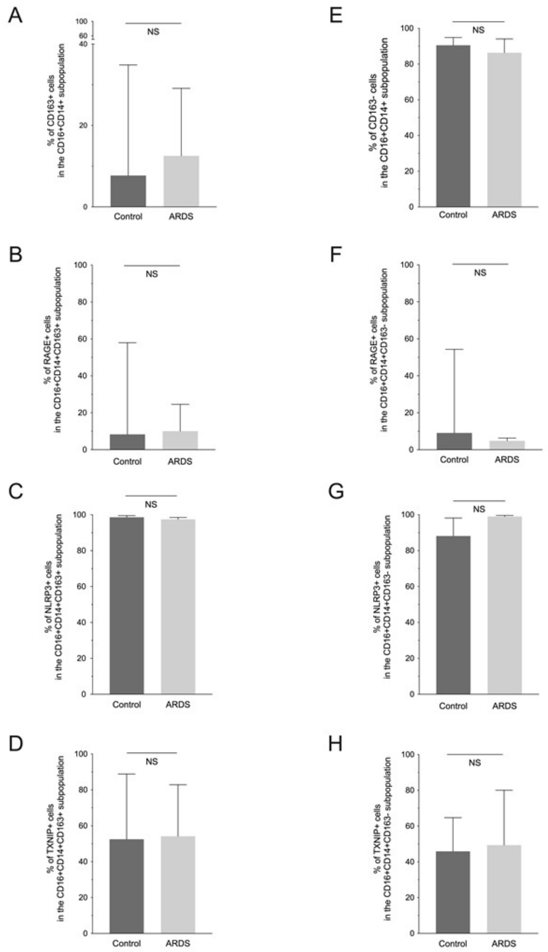

Figure S6. Percentages of **A)** CD163+ cells in the CD16+CD14+ subpopulation, **B)** RAGE+ cells within the CD16+CD14+CD163+ subpopulation, **C)** NLRP3+ cells in the CD16+CD14+CD163+ subpopulation, **D)** TXNIP+ cells in the CD16+CD14+CD163+ subpopulation, **E)** CD163- cells in the CD16+CD14- subpopulation, **F)** RAGE+ cells in the CD16+CD14+CD163- subpopulation, **G)** NLRP3+ cells in the CD16+CD14+CD163- subpopulation, and **H)** TXNIP+ cells in the CD16+CD14+CD163- subpopulation of alveolar macrophages from patients with ARDS and in mechanically ventilated patients without ARDS (control). Values are reported as medians ± interquartile ranges. The data were analyzed using the Mann–Whitney test (nonparametric data).

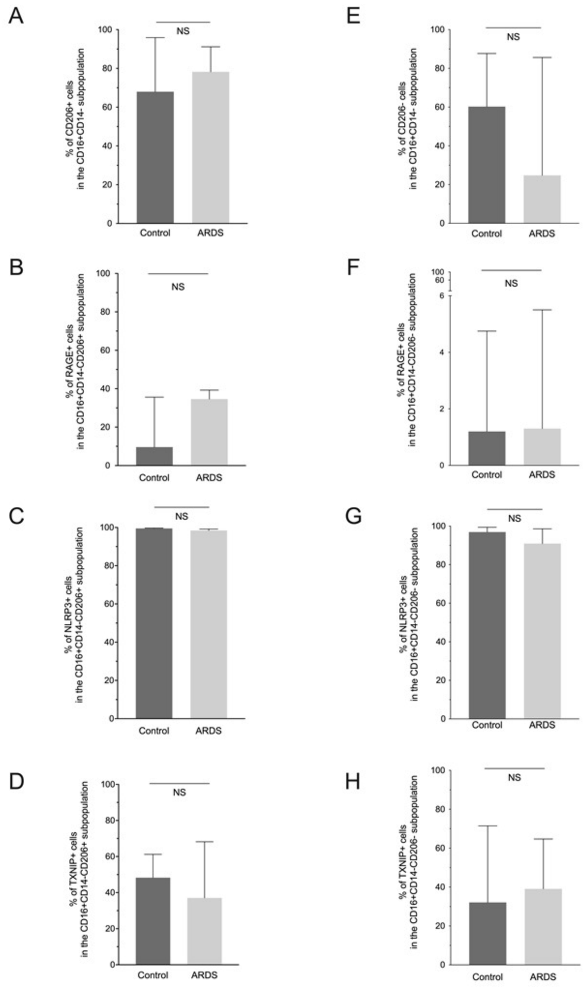

Figure S7. Percentages of **A)** CD206+ cells in the CD16+CD14- subpopulation, **B)** RAGE+ cells in the CD16+CD14-CD206+ subpopulation, **C)** NLRP3+ cells in the CD16+CD14-CD206+ subpopulation, **D)** TXNIP+ cells in the CD16+CD14-CD206+ subpopulation, of **E)** CD206- cells in the CD16+CD14- subpopulation, **F)** RAGE+ cells in the CD16+CD14-CD206- subpopulation, **G)** NLRP3+ cells in the CD16+CD14-CD206- subpopulation, and **H)** TXNIP+ cells in the CD16+CD14-CD206- subpopulation of alveolar macrophages from patients with ARDS and in mechanically ventilated patients without ARDS (control). Values are reported as medians ± interquartile ranges. The data were analyzed using the Mann–Whitney test (nonparametric data).

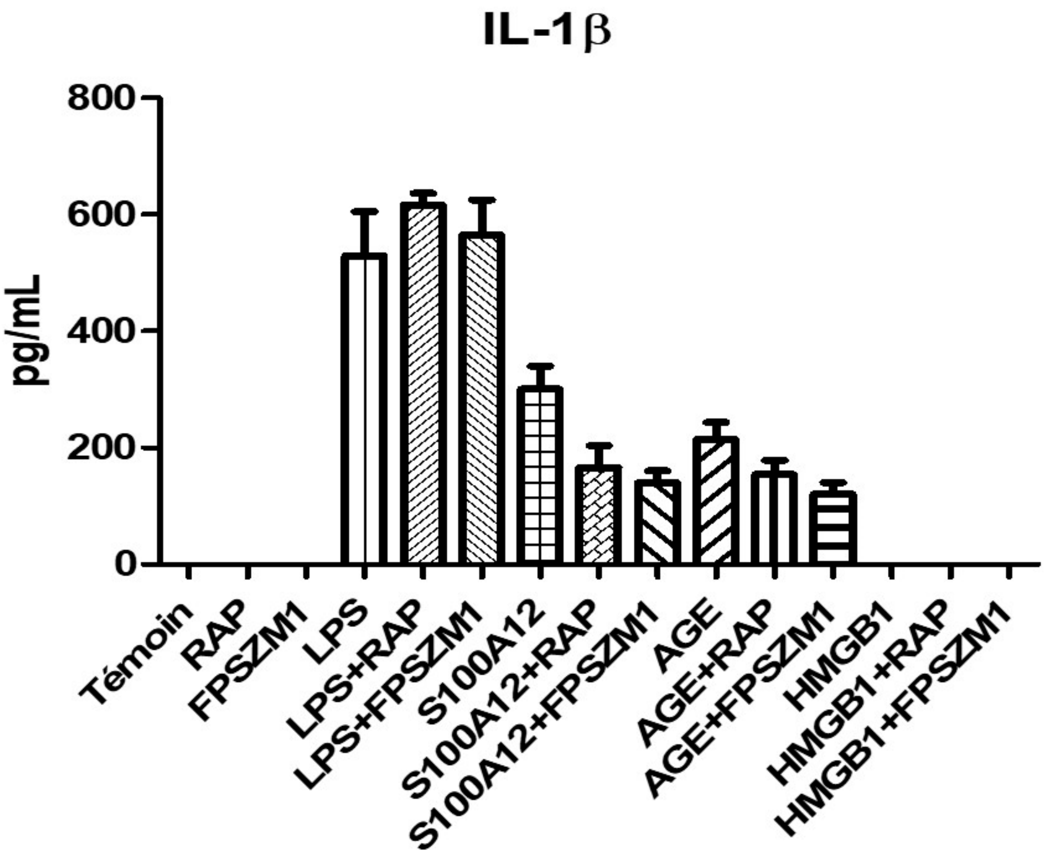

Test RAGE agonist_THP-1_IL1b

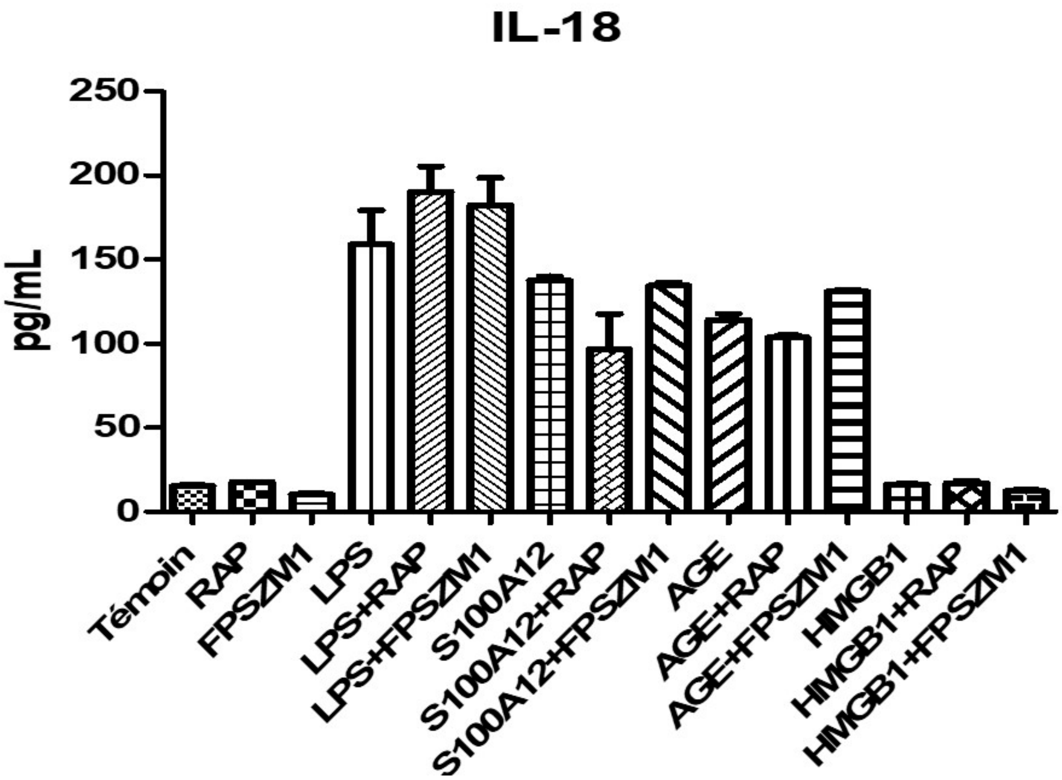

Test RAGE agonist_THP-1_IL18

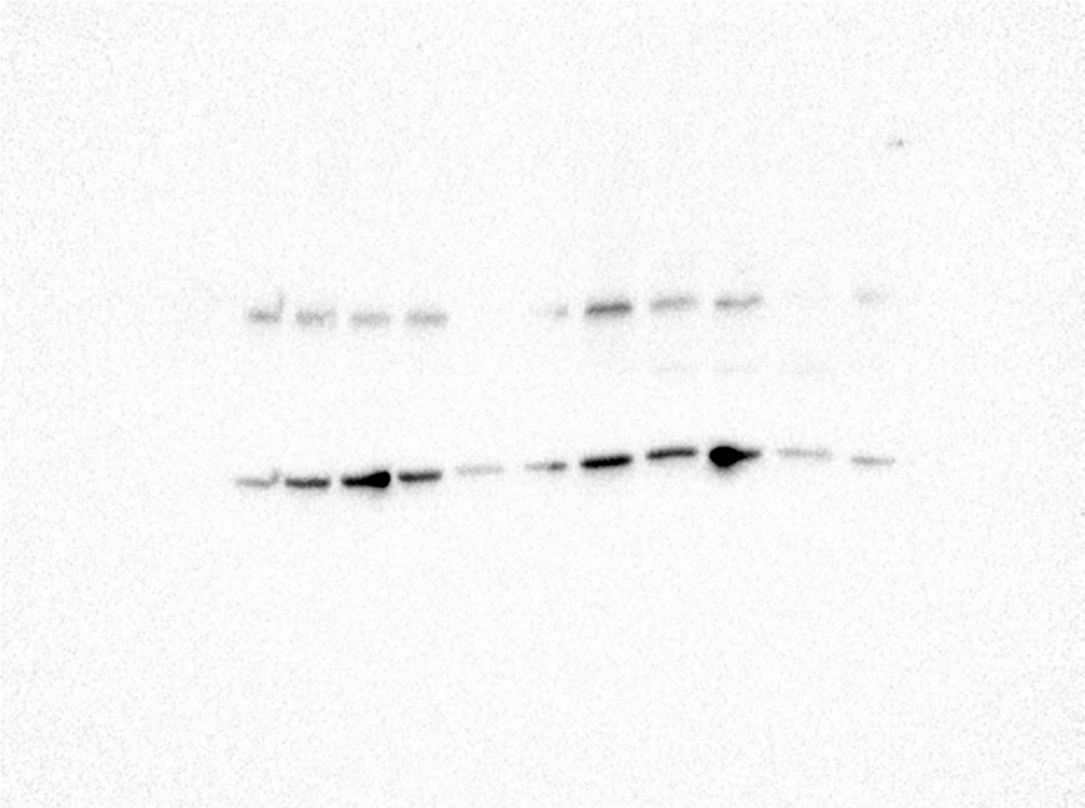

WB_NLRP3_S100A12

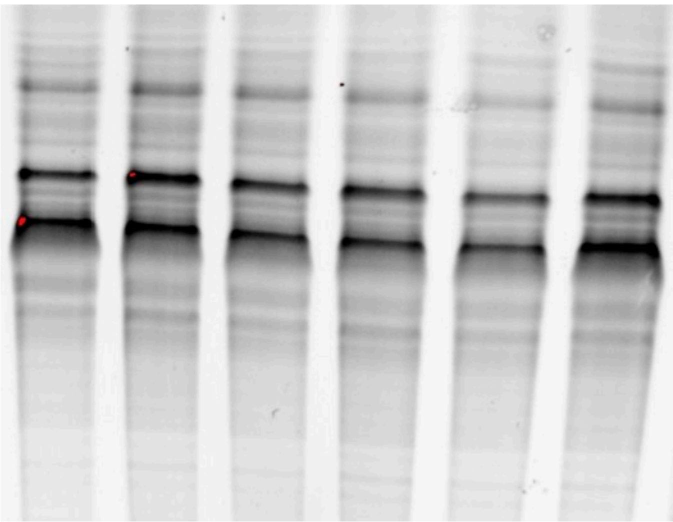

WB_QuantifProt_TXNIP_siRNA

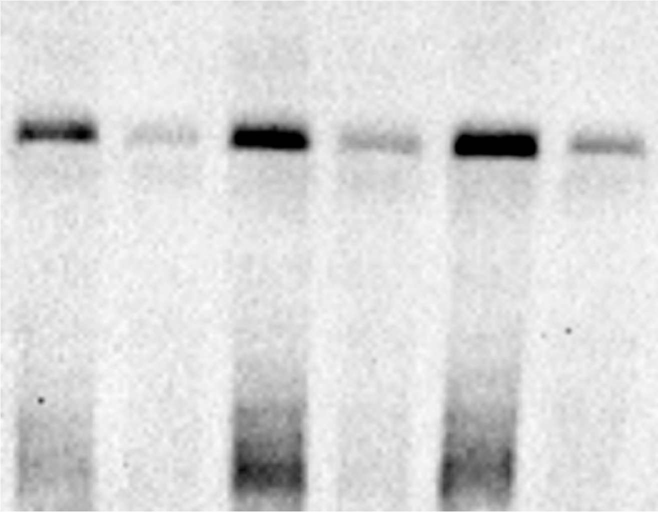

WB_TXNIP_siRNA
